# Non-coding RNA mediates endoplasmic reticulum stress-induced apoptosis in heart disease

**DOI:** 10.1016/j.heliyon.2023.e16246

**Published:** 2023-05-13

**Authors:** Mingyuan Fan, Jing Zhang, Lei Zeng, Danpeng Wang, Jiao Chen, Xiaorong Xi, Jing Long, Jinzhu Huang, Xueping Li

**Affiliations:** a Department of Senile Disease, Hospital of Chengdu University of Traditional Chinese Medicine, Chengdu 610072, China; b Chengdu University of Traditional Chinese Medicine, Chengdu 611137, China

**Keywords:** Apoptosis, Endoplasmic reticulum stress, Non-coding RNA, microRNA, Ischemia/reperfusion, Heart disease

## Abstract

Apoptosis is a complex and highly self-regulating form of cell death, which is an important cause of the continuous decline in ventricular function and is widely involved in the occurrence and development of heart failure, myocardial infarction, and myocarditis. Endoplasmic reticulum stress plays a crucial role in apoptosis-inducing. Accumulation of misfolded or unfolded proteins causes cells to undergo a stress response called unfolded protein response (UPR). UPR initially has a cardioprotective effect. Nevertheless, prolonged and severe ER stress will lead up to apoptosis of stressed cells. Non-coding RNA is a type of RNA that does not code proteins. An ever-increasing number of studies have shown that non-coding RNAs are involved in regulating endoplasmic reticulum stress-induced cardiomyocyte injury and apoptosis. In this study, the effects of miRNA and LncRNA on endoplasmic reticulum stress in various heart diseases were mainly discussed to clarify their protective effects and potential therapeutic strategies for apoptosis.

## 1 Introduction

Cardiovascular disease is the main cause of global mortality. The total prevalence of the cardiovascular disease has almost doubled from 271 million in 1990 to 523 million in 2019, and it is still the main burden in high-income countries [1]. Cardiovascular diseases can be caused by a variety of factors, it has been found that limiting the apoptosis of myocardial and non-cardiomyocytes can effectively protect the heart [2,3]. The characteristics of apoptosis are cell contraction, chromatin condensation, DNA fragmentation, and the formation of apoptotic bodies [4]. For apoptosis, the initiation mechanisms include (1) NF-κB and soluble Fas ligand pathways mediated by the tumor necrosis factor (TNF) receptor gene superfamily; (2) mitochondrial damage; (3) Balance of bcl2-associated X(Bax)/b-cell lymphoma-2(Bcl 2); (4) cysteinyl aspartate specific proteinase (caspase); (5) Mitogen-activated protein kinase(MAPK) signaling pathway [5,6]. Ischemia, hypoxia, inflammatory, and intracellular calcium ion overload is the main factor of apoptosis in the heart [7], while the calcium ion overload and mitochondrial damage induced by endoplasmic reticulum stress is an important mechanism of apoptosis in heart disease [[8], [9], [10]].

Endoplasmic reticulum stress (ERS) often occurs in the ischemic and hypoxic state of cardiomyocytes. Misfolded proteins will affect the resident calcium-dependent chaperone protein and calcium homeostasis of the endoplasmic reticulum. This process is called ERS [11]. In order to ensure correct protein folding and calcium homeostasis, as well as activation of the signal transduction pathway of the unfolded protein response (UPR). As a capping protein, Bip/GRP78 (Glucose-regulated protein 78) will separate from IRE1α (inositol-requiring enzyme 1α), PERK (protein kinase R-like ER kinase), and ATF6 (activating transcription factor 6) to promote their activation [12]. IRE1α is a protein kinase distributed on the endoplasmic reticulum membrane. When ERS occurs, IRE1α cuts the mRNA of X-box binding protein 1 (XBP1) and promotes the translation of XBP1 (sXBP1) after active splicing. Active sXBP1 will promote the translation of the multiple UPR related transcriptome [13]. In addition, IRE1α interacts with TNF receptor-associated factor 2 (TRAF2) and apoptosis signal-regulating kinase 1 (ASK1) to induce c-Jun N-terminal kinase(JNK), p38 MAPK pathways and mediate apoptosis [14]. p38 can also activate enhancer-binding protein homologous protein (CHOP) through phosphorylation of its transactivation domain to inhibit the expression of anti-apoptotic protein Bcl-2 to induce cell apoptosis [5]. The activation of CHOP mediates the transcription of many genes encoding a variety of pro-apoptotic proteins [15]. PERK is also distributed on the endoplasmic reticulum membrane. After phosphorylation, the alpha subunit of translation initiation factor 2 (Eif2α) is phosphorylated to inhibit protein translation. However, long-term ERS causes Eif2α to activate ATF4 transcription and mediate the expression of CHOP/growth arrest and DNA damage-inducible gene 153(GADD153) to promote apoptosis [16]. ATF6 is first phosphorylated and translocated from the ER to the Golgi apparatus and is cleaved into the N-terminal ATF6α, then ATF6α migrates from the cytoplasm to the nucleus, binds to transcription factors and ERS response elements, and is used to transcribe and induce UPR-related transcripts [17].

**Fig. 1:**
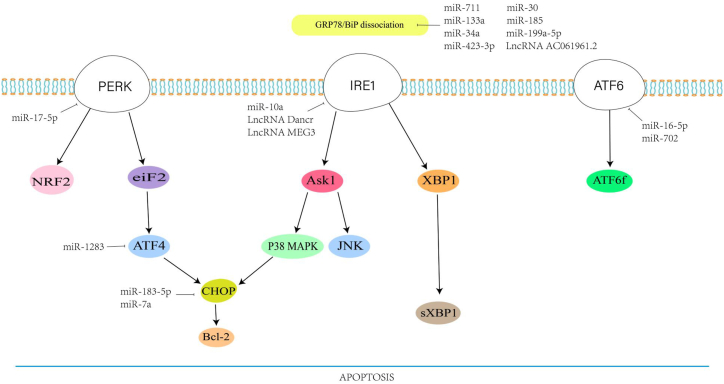
ncRNAs are involved in ERS-mediated apoptosis in the heart.

**Fig. 2:**
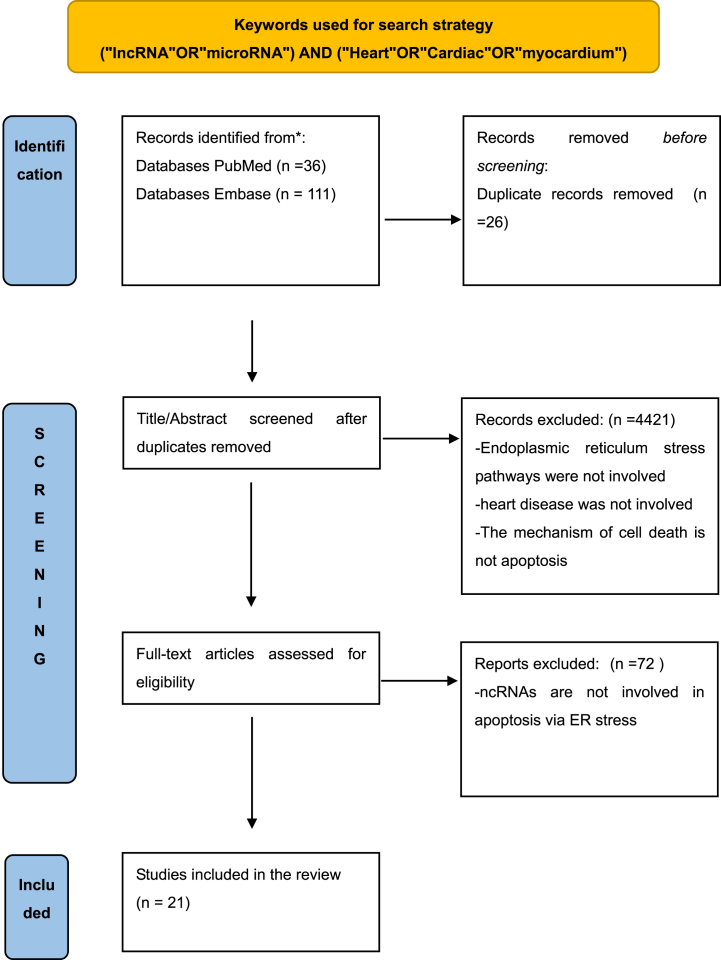
The PRISMA of this study.

Non-coding RNA (including rRNA, tRNA, snRNA, microRNA, etc.) is involved in various signaling transduction processes of cells [18]. We mainly clarify the effects of miRNA and LncRNA on apoptosis in cardiac diseases. MicroRNA (miRNA) is a small non-coding RNA containing about 22 nucleotides. It is involved in the negative effects of post-transcriptional gene expression by binding to the 3′-untranslated region (3′-UTR) of the target transcripts and controlling its translation [19]. Studies in recent years have shown that it participates in a large number of molecular regulations under the UPR signaling pathway and mediates cell apoptosis. Long non-coding RNA (lncRNA, circRNA) is non-coding RNA with a length of more than 200 nucleotides. It has been recognized as an important regulator in various cellular processes. it not only directly participates in the downstream pathways of UPR, but also interacts with miRNAs to form a complex ERS regulation mechanism [20]. For example, the overexpression of lncRNA RPPH1 not only down-regulates GRP78 and CHOP in hippocampal pyramidal neurons, but also directly targets miR-326 to attenuate ERS and apoptosis induced by Aβ25-35 in Alzheimer's disease [21]. In recent years, there is a growing literature that non-coding RNAs have participated in the ERS signaling pathway in cardiovascular diseases [22,23]. The interaction of these non-coding RNAs and their regulatory effects on proteins-mediated apoptosis plays an important role in different heart diseases(Fig. 1). Clarifying their mechanism of action and their regulatory targets contribute to the protection of cardiomyocytes under stress and the effective intervention of apoptotic pathways in cardiac diseases. Thus，we searched the main article databases (PubMed and Embase) with the following keywords: ((“lncRNA” OR “microRNA”) AND (“Heart” OR “Cardiac” OR” myocardium”) AND(“apoptosis” OR “Endoplasmic Reticulum Stress”)) until June 30, 2022. After detailed screening and reading, we Included the miRNA and lncRNA that mediated endoplasmic reticulum stress-induced apoptosis in different cardiac diseases. The search strategy and detailed study selection are presented in Fig. 2.

## 2 MicroRNA regulates ERS-mediated apoptosis

### 2.1 Heart failure

#### 2.1.1 MicroRNA-183-5p and AGGF1

MicroRNA-183-5p mediates various cell proliferation, invasion, and apoptosis. By regulating a variety of molecular mechanisms, it has been discovered that miR 183 can inhibit apoptosis in a number of cancer diseases [24,25]. Duomao Lin et al. discovered for the first time that MiR-183-5p reduces I/R-induced cardiomyocyte apoptosis by targeting voltage-dependent anion channel 1, confirming its beneficial action against cardiovascular diseases [26]. Angiogenic Factor With G-Patch and FHA Domains 1 (AGGF1) is induced by angiotensin II and nuclear factor-κB (NF-κB) to participate in angiogenesis [27]. Recent studies have revealed that AGGF1 can inhibit cardiac cell apoptosis as a result of ischemia-reperfusion [22,28]. AGGF1 regulates the ERS signaling pathway by inhibiting ERK1/2 from activating, this lowers the level of transcription repressor-ZEB1 and promotes the expression of miR-183-5p. The expression of miR-183-5p down-regulated CHOP and inhibited cell apoptosis induced by ERS. The decrease in CHOP expression reduces the negative feedback of growth arrest and DNA damage-inducible gene 34(GADD34), thereby increasing the phosphorylation of eIF2a. Therefore, targeting AGGF1 and miR-183-5p regulation may be a new target for the treatment of heart failure [28].

#### 2.1.2 MicroRNA-939-5p and iNOS/TNFα

The early stages of chronic heart failure(CHF) are accompanied by inflammation-induced aberrant gene expression, followed by myocardial injury and apoptosis as the disease progresses [29]. Aberrant expression of miR-939-5p occurs at different stages of CHF. miR-939-5p regulates the target genes iNOS and tumor necrosis factor-α (TNFα) to inhibit myocardial injury and ERS apoptosis, thereby protecting cardiac microvasculature and slowing down the progression of CHF. Among them, lncRNA-NOS2P3 in turn acts as a sponge RNA to regulate miR-939-5p expression. In the early stage of CHF, lnc-NOS2P3 is lowly expressed to release miR-939-5p, which controls the target genes iNOS and TNFα as well as NO synthesis during New York Heart Association Class (NYHA) I-II phase, thereby inhibiting myocardial and ERS apoptosis. As CHF progresses, inflammatory cytokine burst grows, lnc-NOS2P3 expression increases and downregulates miR-939-5p, and target gene iNOS activation and high levels of TNFα and NO production induce massive myocardial injury and ERS apoptosis, at which point the heart enters NYHA III-IV decompensation. Therefore, regulation of the MiR-939-5p-iNOS/TNFα axis is expected to inhibit the progression of CHF [30].

### 2.2 Myocardial infarction

#### 2.2.1 MicroRNA-155 and SOCS1/NF-κB

MiRNA-155 is considered to be a crucial pleiotropic cell homeostasis regulator, which plays a prominent role in immunity, inflammation, and infection [[31], [32], [33]]. miRNA-155 is significantly down-regulated in coronary artery disease, which may be a prognostic marker of cardiac death in patients after myocardial infarction(MI) [34]. Suppressor of cytokine signaling (SOCS) is an inhibitor of cytokine and growth factor signaling. SOCS is activated by interleukin-6 cytokines in response to pressure overload and ischemic injury, then participates in a complex cascade of signaling for myocardial growth and survival by controlling key negative feedback intracellular pathways [35]. SOCS1 is closely related to ventricular remodeling and apoptosis, such as inhibiting apoptosis induced by TNF-α [36]. Studies have shown that miR-155-5p in the valve directly targets SOCS1 [37]. miR-155 is highly expressed in macrophages in cardiac tissues after MI, acute viral myocarditis, and cardiac hypertrophy. miR-155 expressed by macrophages regulates the function of cardiac fibroblasts and cardiomyocytes through paracrine pathways. This provided a basis for the cardioprotective effect of microRNA-155 [38]. Juan Hu et al. found that the level of miR-155 increased significantly after MI by constructing a rat model of MI. MI increases macrophage infiltration, NF-κB activation, ERS-induced apoptosis, and SOCS1 expression. miR-155 mediates the expression of CHOP, cleaved-caspase-3, caspase-12 by targeting SOCS1 and leads to cardiomyocyte apoptosis after MI [39]. The knockdown of SOCS1 in macrophages promoted hypoxia-induced activation of the NF-κB pathway in neonatal rat cardiomyocytes and ERS-induced cardiomyocyte apoptosis, but inhibition of miR-155 significantly reduced the above effects. This indicates that miR-155 inhibition can attenuate ERS-induced cardiomyocyte apoptosis after MI by reducing macrophage inflammation via the SOCS1/NF-κB pathway [39].

#### 2.2.2 MicroRNA-17-5p and Tsg101

As a potential IRE1α ribonuclease substrate, microRNA-17 (miR-17) was found to control thioredoxin-interacting protein(TXNIP) mRNA expression at the post-transcriptional level [40]. IRE1α induces the denaturation of miR-17 to make TXNIP mRNA more stable to increase the expression level of TXNIP. It has been reported that miR-17 can inhibit hypoxia-induced apoptosis of smooth muscle cells of the kidney, heart, and pulmonary artery [41,42]. Tumor susceptibility gene 101 (Tsg101) is a new cardioprotective agent against oxidative stress triggered by ischemia/reperfusion (I/R) [43]. Overexpression of Tsg101 improves myocardial function during ischemia-reperfusion, and promotes the redistribution of glucose transporter-4 to the muscle membrane, leading to increased glucose entry and adenosine triphosphate (ATP) production in the I/R heart, thereby weakening I/R Induced cardiac dysfunction [44]. Linlin Zhao et al. found that the expression level of miR-17-5p in MI/R mice and hypoxic cell models was significantly increased, accompanied by increased apoptosis. Inhibition of miR-17-5p resulted in the reduction of the expression and apoptosis of the biomarkers, such as Bip, CHOP, and caspase-12, as well as caspase-3 cleaved by pro-apoptotic factors in the hypoxia model. In addition, 4-Phenylbutyric acid (4-PBA), an inhibitor of ERS response, eliminated apoptosis induced by the rise of shTsg101 in hypoxic cardiomyocytes. This reveals the miR-17-5p/Tsg101 regulatory axis in ERS-induced myocardial injury and cardiomyocyte apoptosis [45].

#### 2.2.3 MicroRNA-711 and PPARγ

In I/R-injured heart cells, miR-711 was significantly up-regulated. The introduction of exogenous miR-711 into cells aggravated cell apoptosis/death and mitochondrial damage. MiR-711 exacerbated cell damage caused by oxidative stress, such as H/R, by suppressing the production of Angiopoietin-1, FGF14, and Cacna1c [46]. Overexpression of miR-711 can promote the expression of NF-κB (p65) in ischemia-reperfusion cardiomyocytes, accelerate the transport of NF-κB (p65) to the nucleus, and promote the apoptosis of cardiomyocytes [47]. Peroxisome proliferator-activated receptor γ (PPARγ), as a ligand-activated nuclear receptor, is widely involved in regulating glucose and lipid metabolism, endothelial function, and inflammation [48]. Na Zhao et al. found that PPARγ upregulates miR-711 in adipocytes and cardiac fibroblasts. Transfection of miR-711 mimics into cardiomyocytes can induce endoplasmic reticulum (ER) stress-mediated apoptosis. On the second day after the MI model was constructed, it was found that the increase in the level of miR-711 in the heart was accompanied by an increase in cardiomyocyte apoptosis, a decrease in the level of calnexin, and the levels of GRP78, ATF6, sXBP1, ASK1, CHOP, and caspase-12 increase, and apoptosis of cardiomyocytes. This is due to the fact that PPARγ promotes miR-711 expression by directly binding to the pre-miR-711 promoter, and miR-711 regulates ER stress-mediated cardiomyocyte apoptosis through calpain. These discoveries add to the treatment strategies for early heart remodeling after MI and provide potential therapeutic targets [49].

#### 2.2.4 MicroRNA-7a and CHOP

In cardiovascular disease, miR-7 was first reported to be associated with the risk of coronary artery disease and was upregulated in cardiomyocytes during I/R [50]. Subsequent studies found that miR-7a/b can protect cardiomyocytes from apoptosis during I/R injury by targeting Poly (ADP-Ribose) Polymerase 1 (PARP-1) [51]. Hypoxia triggers the activation of the binding activity of Sp1 with PARP-1 and caspase-3 promoters, which can be directly inhibited by miR-7a/b to improve cardiac function, weaken cardiac remodeling, and reduce fibrosis and apoptosis [52]. By inducing ERS and apoptosis in embryonic rat cardiac myoblasts H9c2, it was found that the imbalance of miR-7a was the most significant. Glucose deprivation and hypoxia induced the expression of GRP78 and HERP(UPR target genes), and elevated the levels of miR-7a. In numerous cell lines, CHOP overexpression promotes apoptosis [[53], [54], [55]]. By eliminating the induction of CHOP, miR-7a overexpression provides resistance to apoptosis induced by ER stress [56].

### 2.3 Ischemic heart disease (I/R, H/R)

#### 2.3.1 MicroRNA-133a and H2S

MicroRNA-133a (miR-133a) is one of the most abundant miRNAs in the heart [57]. It has shown that miR-133a is involved in the early pathological process of MI and subsequent ventricular remodeling, and can be combined with NT-proBNP as a diagnostic marker for MI [58]. Furthermore, miR-133 can mediate the expression of cardiomyocyte-associated apoptotic proteins Bcl-2, Bax, and caspase-9 [58]. Lin Ren et al. used H2S combined with miR-133a overexpression to treat I/R-induced cardiomyocytes, revealing that H2S reduces ER stress and apoptosis by regulating the expression of miR-133a. The cardioprotective effect of miR-133a overexpression provides a new therapeutic method for the treatment of ischemic heart disease [59].

#### 2.3.2 MicroRNA −34a and Sirt1/Nrf2

MicroRNA −34a is induced in aging hearts, and the silencing/absence of miR-34a reduces age-related cardiomyocyte death [60]. Inhibition of miR-34a can reduce cell death and fibrosis after acute myocardial infarction, and improve the recovery of myocardial function [61]. Studies have demonstrated that miR-34a can directly down-regulate vascular smooth muscle SIRT1, inhibit cell proliferation, and induce senescence, consequently promoting vascular calcification [62]. Xiaowu Wang et al. constructed an I/R mouse model and found that crocin reduced the expression of miR-34a, up-regulated sirtuin 1(Sirt1), nuclear factor erythroid-2 related factor 2(Nrf2), and HO-1 levels, and significantly reduced I/R-induced cardiomyocytes apoptosis. Nrf2 is a classic PERK downstream molecule, which indicates that regulating the miR-34a/Sirt1/Nrf2 pathway helps protect cardiomyocytes from ERS and apoptosis induced by I/R injury [63].

#### 2.3.3 MicroRNA -423-3p and ERK

Activation of the inflammasome in cardiac fibroblasts (CFs) mediates I/R injury in myocardium under Hypoxia/reoxygenation (H/R) stimuli [64]. H/R significantly increases the exosomes/microvesicle secretion of CFs, among which the expression of 423-3p was significantly up-regulated [65]. In a 6-year follow-up diagnostic clinical trial of coronary artery disease, miR-423-3p can accurately predict the occurrence of the disease [66]. The extracellular regulated protein kinases(ERK) signaling pathway is a key regulatory pathway that regulates ER-induced apoptosis and I/R damage [67]. TIAN-RUI YANG et al. constructed an I/R model and discovered that miR-423-3p reduced the expression of ERK and the expression of ERS markers GRP78 and CHOP respectively, thereby reducing cardiomyocyte apoptosis. This demonstrated the cardioprotective effect of miR-423-3p [68].

#### 2.3.4 MicroRNA −93 and PTEN

Recent studies have found that miR-93 exerts anti-inflammatory and anti-apoptotic effects in the pathological process of cardiomyocyte dysfunction. It regulates apoptosis and inflammation by targeting signal transducer and activator of transcription 3 (STAT3), Bcl-2, and toll-like receptor 4(TLR4) [69,70]. Phosphate and tension homology deleted on chromosome ten (PTEN) negatively regulates the activation of phosphatidylinositol 3-kinase (PI3K)/Akt signaling pathway and leads to cardiomyocyte apoptosis [71]. In the H/R model, pretreatment of miR-93 significantly inhibited GRP78, CHOP, caspase-12 levels, and ROS generation. The 3′-UTR of PTEN mRNA contains a miR-93 binding site, which can be directly targeted. Therefore, the inhibition of *p*-Akt in H/R can be improved by miR-93 [72]. Overexpression of miR-93 is the reason for reducing ERS-related cell apoptosis and protecting cardiomyocytes during I/R injury.

#### 2.3.5 MicroRNA −10a and IRE1

MicroRNA −10a is a member of the miR-10 family and has been shown to play a direct role in the proliferation and differentiation of many cell types [[73], [74], [75]]. Recent studies have shown that miR-10a is closely related to the occurrence and development of cardiovascular-related diseases [76], like mediating TGF-β1/Smads signaling to regulate cardiac fibrosis in atrial fibrillation [71], also been suggested as a biomarker for the prognosis of heart failure [77]. In the H/R model, miR-10a is highly expressed, H/R injury leads to a decrease in the expression ratio of Bcl-2 and Bax, as well as considerable downregulation of GRP78, IRE1, and XBP1. The miR-10a inhibitor reversed the above results. qPCR detection showed that the expression of IRE1 mRNA was not affected, suggesting that IRE1 is post-transcriptionally regulated by miR-10a. This reveals that miR-10a promotes myocardial I/R injury by down-regulating the ERS-related protein IRE1, which provides a theoretical basis for preventing I/R myocardial injury and apoptosis [78].

### 2.4 Ischemic dilated cardiomyopathy (ICM)

#### 2.4.1 MicroRNA -16-5p and ATF6

Ischemic dilated cardiomyopathy (ICM) is the most prevalent cause of dilated cardiomyopathy(DCM) and leads to malignant arrhythmias and heart failure and is thus considered one of the most common causes of heart transplantation [79]. It was found that microRNA-16-5p is upregulated at the expression level of ICM, which induces apoptosis in cardiomyocytes. microRNA-16-5p directly targets ATF6 downregulation and inhibits ATF6-mediated endoplasmic reticulum stress-induced cytoprotective response, leading to mitochondrial dysfunction and increased ROS production, promoting cardiomyocyte inflammation and apoptosis [80]. In addition, miR-16-5p overexpression promoted ER stress cascade responses, including upregulation of the PERK/CHOP pathway, and downregulation of the anti-apoptotic protein Bcl-2, which would increase cellular sensitivity to apoptosis [81].

### 2.5 Another miRNAs

#### 2.5.1 Cardiomyocytes—miR-199a-5p and GRP78/ATF6

Chronic hypoxia of cardiomyocytes is a common pathophysiological process of coronary heart disease, cyanotic congenital heart disease (CCHD), and many other cardiovascular diseases [82]. miR-199a-5p is the cause of the pathophysiological changes that lead to and promote the development of heart disease. The level of miR-199a-5p will rapidly decrease in hypoxic myocardium, by promoting its target genes-hypoxia-inducible factor (HIF), vascular endothelial growth factor (VEGF) at the post-transcriptional level to regulate myocardial oxygen metabolism and cardiac angiogenesis and protect the hypoxic-ischemic myocardium [83]. Yang Zhou et al. constructed a mouse model under hypoxic conditions and found that hypoxia-induced a significant decrease in the level of miR-199a-5p in mouse myocardial tissue, and inhibition of signal transducer and activator of transcription 3(STAT3) phosphorylation could significantly prevent the decrease of miR-199a-5p. MiR-199a-5p is derived from two different original microRNAs, namely pri-miR-199a-1 and pri-miR-199a-2 [84]. Hypoxia and activation of STAT3 mainly affect the expression of pri-miR-199a-2, but have no significant effect on the expression of pri-miR-199a-1. Chronic hypoxia can down-regulate the level of miR-199a-5p by activating the STAT3 pathway, and reducing miR-199a-5p will promote the expression of GRP78 and ATF6, which is beneficial to the UPR process and protects cardiomyocytes from ERS-related apoptosis. Therefore, STAT3 and miR-199a-5p may be valuable diagnostic and therapeutic targets for patients with chronic hypoxic heart disease [85].

#### 2.5.2 Cardiomyocytes—miR-185 and NHE-1

The Na^+^/H^+^ exchanger isoform-1 (NHE-1) is a plasma membrane glycoprotein responsible for the main Na + influx pathway in heart cells. NHE-1 is the main subtype in the myocardium [86]. Recent studies have confirmed the beneficial effects of NHE inhibition in protecting the myocardium from ischemia and reperfusion injury, as well as reducing myocardial remodeling and heart failure [87]. miR-185 plays an important anti-hypertrophic effect in the heart through multiple targets of Ca2^+^ signaling and participates in the process of angiogenesis and heart function recovery under hypoxic conditions [88]. In recent years, it has been found that miR-185 directly targets the 3′- untranslated region of Na^+^/H^+^ exchanger −1(NHE-1), which significantly reduces the expression of pro-apoptotic proteins(CHOP, cleaved-caspase-3) in cardiomyocytes induced by ERS in a dose-dependent manner. This suggests that miR-185 and NHE-1 are involved in ERS-mediated apoptosis [89].

#### 2.5.3 Cardiomyocytes—miR-702 and ATF6

MiR-702 derived from the 13th intron of the mouse Plod 3 gene: mmu-mir-702. Although it has been demonstrated that the interaction between ATF63′-UTR binding site in mouse myocardium can reduce apoptosis, miR-702 has a low expression level in humans, making it necessary to further investigate whether it has the same anti-myocardial apoptotic effects in humans [90].

#### 2.5.4 Cardiac vascular endothelial cells—miR-1283 and ATF4

Vascular endothelial cells have a highly developed endoplasmic reticulum and are very sensitive to ERS [91]. Recent research has shown that ATF4 is highly expressed in damaged vascular endothelial cells and may be the target of miR-1283 [92]. Ling He et al. have reported that the inhibition of miR-1283 increases the apoptosis of cardiac tissue. In addition, the expression of apoptosis-related protein BAX and GPR78 also increased. Inhibited the overexpression of miR-1283 or ATF4 promoted the damage of vascular endothelial cells in heart tissue. Inhibition of miR-1283 enhances ERS by preventing ATF4 from regulating the PERK/ATF4 pathway, causing apoptosis and inflammation, and eventually vascular endothelial damage [93]. In addition, recent studies have found that growth arrest and DNA damage inducible protein Alpha(GADD45A)plays an important regulatory role in various cell functions, such as DNA repair, cell cycle regulation, and genotoxic stress response [94]. GADD45A was identified as a downstream target of miR-1283 and was upregulated in MI. MicroRNA1283 can reduce cardiomyocyte damage caused by H/R by targeted GADD45A to inactivate JNK and p38 MAPK signaling pathways. This reveals the important role of miR-1283 in inducing apoptosis in the ischemic environment [95].

#### 2.5.5 Vascular smooth muscle cells and cardiomyocytes—miR-30 and GRP78

The miR-30 family includes five members: miR-30a, miR-30b, miR-30c, miR-30d, and miR-30e. They have common precursors with highly conserved sequences. Studies have shown that miR-30c can be targeted peroxlsome proliferator-activated receptor-γ coactlvator-1β(PGC-1β) to reduce myocardial cell apoptosis in diabetic cardiomyopathy [96]. The miR-30a, b, c, d, and e of the miR-30 family are all down-regulated in ER stress-induced by H2O2-induced ventricular cells and aortic vascular smooth muscle cells, accompanied by up-regulation of GRP78, lytic ATF6, CHOP, and cleavage Caspase-12. The down-regulation of miR-30 family miRNA contributes to the up-regulation of GRP78 in the ERS and cardiovascular system. This indicates that the involvement of miR-30 in the ERS signaling pathway produces a positive feedback mechanism [97]. However, recent studies have revealed that in renal tubular cells, MiR-30a-5p is a true negative regulator of GRP78 expression, while MicroRNA-30c-2-3p regulates ERS and induces ovarian cancer cell apoptosis [98,99]. Therefore, the specific mechanism of the miR-30 family's effect on ERS in vascular smooth muscle cells and cardiomyocytes may require further verification.

## 3 LncRNA regulates ERS-mediated apoptosis

### 3.1 Myocardial infarction

#### 3.1.1 LncRNA Dancr and microRNA-6324

LncRNA Dancr is a new cancer regulator and widely involved in breast cancer, gastric cancer, and other diseases. It is also involved in ceRNA to adsorb various miRNAs to act on tumor growth, drug resistance, and other links [100,101]. In recent years, studies have found that DANCR has a negative regulatory effect on cardiomyocytes under hypoxic conditions. Overexpression of DANCR attenuates apoptosis and promoted the activation of PI3K/AKT/mammalian target of rapamycin(mTOR) and ERK1/2 pathways [102]. In MI model rats, microRNA-6324 is up-regulated and is predicted to bind to Dancr. Dancr overexpression significantly down-regulated the expression of miR-6324 and Bcl-2, and the expression level of Bax and cleaved-caspase-3/9 increased significantly. Dancr selectively promotes the phosphorylation of IRE1α and activates the IRE1α branch in UPR. In addition, miR-6324 mimic partially reversed the apoptosis, ERS, and autophagy caused by Dancr overexpression. This indicates that overexpression of lncRNA Dancr protects cardiomyocytes from ERS damage through sponge miR-6324, thereby inhibiting apoptosis and restoring ER homeostasis [23].

#### 3.1.2 LncRNA MEG3 and p53

Human maternally expressed gene 3 (MEG3) is a tumor suppressor lncRNA that regulates the p53 pathway. In most primary tumors, the hypermethylation of the maternal allele of MEG3 is down-regulated, and its ectopic expression will reduce tumor progression; Therefore, MEG3 usually acts as a tumor suppressor [103,104]. In adult cells, MEG3 stimulates the p53 pathway and induces cell cycle arrest and apoptosis [105]. In recent years, its role in the cardiovascular system has become more abundant, and it has participated in various diseases and pathological processes such as myocardial fibrosis, myocardial hypertrophy, and mitochondrial-mediated apoptosis [106,107]. Xueling Li et al. established MI mice and found that at 28 days after MI, lncRNA MEG3 knockdown mice showed better heart function and less heart remodeling. Knockdown of lncRNA MEG3 reduced cardiomyocyte apoptosis and the production of reactive oxygen species in ventricular cells of MI mouse models and hypoxic mice. Knockdown of lncRNA MEG3 inhibits ERS through the induction of PERK-eIF2α and caspase 12 pathways after MI. p53 was identified as a protein target of lncRNA MEG3 to regulate NF-κB and ERS-related apoptosis. This clarifies that lncRNAMEG3 knockdown plays a cardioprotective effect by targeting p53 to reduce ERS-mediated apoptosis after MI [108].

### 3.2 Dilated cardiomyopathy

#### 3.2.1 LncRNA AC061961.2 and Wnt/β-catenin pathways

LncRNA AC061961.2 was first screened by Qiu Z et al. Co-expression network analysis of DCM gene matrix and real-time PCR confirmed it was significantly down-regulated in DCM [109]. Subsequently, the expressions of AC061961.2, β-catenin, Axin2, c-Myc, and Bcl-2 were decreased in the DCM model rats established by doxorubicin, and the expressions of CRP78, CHOP, Caspase-3, and Bax were increased. The levels of β-catenin, Axin2, c-Myc, and Bcl-2 increased after AC061961.2 overexpression, while the levels of CRP78, CHOP, Caspase-3, and Bax decreased. This reveals that the overexpression of LncRNA AC061961.2 inhibits ERS-induced apoptosis in DCM rats and cardiomyocytes [110].

### 3.3 Ischemic heart disease

#### 3.3.1 LncRNA UCA1

LncRNA UCA1 has been found to be overexpressed in various human cancers. It has been proven to promote the proliferation, migration, invasion, and immune escape of various tumor cells [111,112]. In recent years, studies have shown that it can bind a variety of proteins or miRNAs to play a cardioprotective or pro-apoptotic effect, and it is recommended as a new biomarker for acute myocardial infarction [113]. Jing Chen et al. triggered the expression of lncRNA UCA1, the generation of ROS in cells and mitochondria, and induced ERS and mitochondrial oxidative stress to induce apoptosis by establishing a H/R model. The overexpression of UCA1 inhabited the expression of GRP78, ATF6 and PERK and apoptosis induced by I/R. Inhibition of ERS reversed the intracellular ROS generation and mitochondrial dysfunction induced by siUCA1. This indicates that the expression of UCA1 prevents I/R-induced oxidative stress and mitochondrial dysfunction by inhibiting ERS [114].

## 4 Concluding statemen

ER stress can activate heart remodeling and myocardial ischemia, the inhibition of ER stress helps protect myocardial cells from stress and enhances resistance to apoptosis. In summary, miRNA and LncRNA directly or indirectly regulate ERS, and some non-coding RNAs directly act on proteins located on the ER membrane and related proteins of UPR downstream signalings. miR-133a, miR-423-3p, miR-10a, and LncRNA UCA1 are recommended as prognostic or diagnostic biomarkers for Ischemic heart disease are, and microRNA-133a is one of the most abundant in cardiomyocytes. These RNAs have the most potential as a therapeutic target. miR-7a, miR-1283, and miR-30 act on the apoptotic pathways by directly targeting ERS-related components, and their effects on ERS-induced apoptosis are definite, these miRNA show potential for further research in specific cardiac diseases. Both miR-183-5p and miR-185 are related to cell ion exchange function, which is of great significance to cardiomyocytes that rely on calcium ion flow to regulate homeostasis. LncRNA AC061961.2 is currently the only non-coding RNA that has been clearly interfered with ERS in dilated cardiomyopathy. Overall, this article highlights the impact of non-coding RNAs mediated ERS on apoptosis in cardiac cells, and they have different characteristics and mechanisms of action (as shown in Table 1, Table 2), and interventions for these non-coding RNAs are beneficial in the treatment of heart diseases.

**Table 1:** The role of microRNAs on the endoplasmic reticulum stress in cardiac disease.

Disease	miRNA	Expression	Target	Pathophysiological effects	Reference
Heart failure	miR-183-5p	Increase	AGGF1 Upstream	Directly inhibit cardiomyocyte apoptosis	Yao et al. [28]
Heart failure	miR-939-5p	Increase	iNOS/TNFα Upstream	Directly inhibit cardiomyocyte apoptosis	Chen et al. [30]
Myocardial infarction	miR-155	Decrease	SOCS1 Downstream	Inhabit the inflammatory response induced by macrophages	Hu et al. [39]
Myocardial infarction	miR-17-5p	Increase	Tsg101 Downstream	Directly inhibit cardiomyocyte apoptosis	Zhao et al. [45]
Myocardial infarction	miR-711	Decrease	PPARγ Downstream	Reduce ischemia reperfusion injury	Zhao et al. [49]
Myocardial infarction	miR-7a	Increase	CHOP Downstream	Directly inhibit cardiomyocyte apoptosis	Read et al. [56]
Ischemic heart disease (I/R; H/R)	miR-133a	Increase	H2S Upstream	Directly inhibit cardiomyocyte apoptosis	Ren et al. [59]
Ischemic heart disease (I/R; H/R)	miR-34a	Decrease	Sirt1/Nrf2 Downstream	Reduce ischemia reperfusion injury	Wang et al. [63]
Ischemic heart disease (I/R; H/R)	miR-423-3p	Increase	ERK Downstream	Directly inhibit cardiomyocyte apoptosis	Yang et al. [68]
Ischemic heart disease (I/R; H/R)	miR-93	Increase	PTEN Downstream	Reduce ischemia reperfusion injury	Ke et al. [72]
Ischemic heart disease (I/R; H/R)	miR-10a	Increase	IRE1 Downstream	Reduce ischemia reperfusion injury	Zhang et al. [78]
Ischemic dilated cardiomyopathy	miR-16-5p	Decrease	ATF6 Downstream	Directly inhibit cardiomyocyte apoptosis	TORO et al. [80]
Cyanotic congenital heart disease	miR-199a-5p	Increase	STAT3 Upstream	Directly inhibit cardiomyocyte apoptosis	Zhou et al. [85]
Cardiomyocytes	miR-185	Increase	NHE-1 Downstream	Anti-cardiac hypertrophy	Kim et al. [89]
Cardiomyocytes	miR-702	Increase	ATF6 Downstream	Not clear	Zhang et al. [90]
Cardiac vascular endothelial cells	miR-1283	Increase	ATF4 Downstream	Reduce ischemia reperfusion injury	He et al. [93]
Vascular smooth muscle cells and cardiomyocytes	miR-30	Increase	GRP78 Downstream	Reduce apoptosis of vascular smooth muscle cells and cardiomyocytes	Chen et al. [97]

**Table 2:** The role of LncRNAs on the endoplasmic reticulum stress in cardiac disease.

Disease	LncRNA	Expression	Target	Pathophysiological effects	Reference
Myocardial infarction	Dancr	Down-regulation by targeting IRE1α	microRNA-6324/IRE1α	Directly inhibit cardiomyocyte apoptosis and maintain calcium homeostasis	Li et al. [23]
Myocardial infarction	MEG3	Decrease	P53	Directly inhibit cardiomyocyte apoptosis	Li et al. [108]
Dilated cardiomyopathy	AC061961.2	Increase	Wnt/β-catenin	Directly inhibit cardiomyocyte apoptosis	Qiu et al. [110]
I/R	UCA1	Increase	Unclear	Alleviate I/R and oxidative stress injury.	Chen et al. [114]

## Author contribution statement

All authors listed have significantly contributed to the development and the writing of this article.

## Funding statement

This work was supported by the National Natural Science Foundation of China [81603615].

## Data availability statement

No data was used for the research described in the article.

## Declaration of competing interest

The authors declare the following financial interests/personal relationships which may be considered as potential competing interests: Xueping Li reports financial support and article publishing charges were provided by National Natural Science Foundation of China.
